# Rapid Strength Prediction of HTV Silicone Rubber Composite Insulators Based on Aging Characteristics

**DOI:** 10.3390/polym18091084

**Published:** 2026-04-29

**Authors:** Zhijin Zhang, Yao Shen, Shude Jing, Jun Deng, Xingliang Jiang, Yutai Li

**Affiliations:** 1 Xuefeng Mountain Energy Equipment Safety National Observation and Research Station, School of Electrical Engineering, Chongqing University, Huxi Campus, Chongqing 400044, China; shenyao@stu.cqu.edu.cn (Y.S.); jingshude@stu.cqu.edu.cn (S.J.); xljiang@cqu.edu.cn (X.J.); liyt@cqu.edu.cn (Y.L.); 2 Southern Power Grid EHV Transmission Company Electric Power Research Institute, Guangzhou 510663, China; dengjun@ehv.csg.cn

**Keywords:** composite insulation parts, molecular simulation, high temperature vulcanized silicone rubber, mechanical aging

## Abstract

To investigate the inevitable aging of composite insulators under the coupled effects of electrical, thermal, ice, and fog stresses, as well as to explore their aging mechanisms and residual strength prediction methods, this study collected operational insulator samples from four environmental regions: Tibet, Yunnan, Hunan Xuefeng Mountain, and Anhui/Chongqing. Mechanical properties, including tensile strength, elongation at break, and shear resistance, were tested. The results indicate that the degradation of mechanical performance in composite insulation components can be attributed to the synergistic interaction of operational environments and material characteristics, with the aging behavior of high-temperature vulcanized (HTV) silicone rubber exhibiting significant non-linearity. Based on existing research, molecular dynamics simulations were employed to construct microstructural models at different aging stages, and it was verified that main chain scission, reduced system density, and changes in the elemental chemical environment during aging are closely related to the degradation of material mechanical properties. Based on hyper-elastic constitutive theory and fracture mechanics, a quantitative method for assessing the comprehensive aging degree was proposed, with “service years” and “operational altitude” as the core dimensions. A negative exponential model was established to describe the strength degradation of silicone rubber materials. This model enables the non-destructive estimation of the residual mechanical strength of in-service insulators in complex regions without power interruption, providing a decision-making framework for grid operation and maintenance.

## 1. Introduction

Silicone rubber is widely used in composite insulators for power systems due to its excellent thermal stability, weather resistance, hydrophobicity, and electrical insulation [1,2]. Currently, over ten million such insulators are in service in China. However, during long-term outdoor operation, they endure not only continuous electric field stress but are also exposed to multiple environmental factors, including temperature extremes, strong UV radiation, and surface discharge [3]. This synergistic stress initiates chemical reactions such as molecular chain session and side-group oxidation, which are followed by filler-matrix interface failure and the evolution of organic components, ultimately leading to a decline in the material’s mechanical properties [4,5].

Extensive research has been conducted worldwide on the aging of composite insulating materials. On the experimental side, many studies have focused on characterizing the macroscopic properties of composite insulators. Reference [6] conducted thermogravimetric analysis and mechanical testing on silicone rubber insulators aged for 2000 h. It revealed that low-molecular-weight substances (approximately 15% of the composition) enable hydrophobicity recovery. The study reported a hardness increase of about 20%, alongside decreases of 30% in tensile strength and 25% in elongation at break. Reference [7] tested silicone rubber aged for 1000 h under UV-B 313 lamps. Prolonged aging gradually decreased tensile strength (rate decelerating), increased then decreased tear strength, and steadily raised hardness (growth slowing). UV radiation breaks C-H and Si-C bonds; the resulting free radicals form cross-links, increasing molecular weight and stress-concentration points, which raises hardness but reduces tensile strength. Reference [8] characterized a nano-SiO_2_/micro-Al_2_O_33_ filled HTV silicone rubber composite after 5000 h of combined aging via XPS, FTIR, TGA, and mechanical tests. The composite with 2% nano-SiO_2_ and 10% micro-Al_2_O_3_ demonstrated optimal performance, achieving a HC-3 hydrophobicity class, the lowest leakage current (3.1 uA), and the least mechanical strength loss (31.3%), alongside good thermal stability. XPS analysis revealed surface oxidation, indicated by a significant increase in oxygen and a corresponding decrease in silicon content. Reference [9] investigated the aging of silicone rubber using SEM, FTIR, and TGA, showing that multiple aging factors alter its surface morphology. The continuous degradation of PDMS chains reduces hydrophobic functional groups, thereby decreasing hydrophobicity. Reference [10] used molecular dynamics and density functional theory simulations to show that high temperature enlarges the free volume at the sheath-core interface and inhibits hydrogen bonding, thereby weakening interfacial adhesion. Reference [11] demonstrated through molecular dynamics simulations that enhanced intermolecular interactions in methyl vinyl silicone rubber suppress local structural motion and restrict molecular chain rotation and diffusion, thereby increasing internal energy dissipation during deformation and improving the material’s macroscopic damping performance. Reference [12] employs molecular dynamics simulations to study silicone rubber. The study reveals that as material performance degrades due to molecular bond cleavage, the microstructure undergoes a progressive evolution, which consequently leads to a reduction in its rigidity.

Experimental research based on macroscopic parameters such as hardness and tensile strength has established a solid qualitative foundation for material aging [6,7,8,9,13,14]. To break through the limitations of qualitative description and achieve quantitative mechanistic analysis, it is crucial to conduct an in-depth investigation into the intrinsic microstructure of the material. Molecular simulation, which can verify and visualize experimentally inferred mechanisms at the atomic scale, bridge the gap between macroscopic performance and microscopic origins [15]. It must be emphasized that the insights gained from simulation are similarly constrained by their spatiotemporal scales and model simplifications.

Composite insulator samples from regions with intensely coupled environmental stresses (electrical, UV, damp-heat, icing)—Tibet, Yunnan, Hunan Xuefeng Mountain, Anhui, and Chongqing—were investigated via integrated experimental methods. By analyzing the macro properties (hardness, tensile strength) and micro defects of aged samples, key aging indicators were extracted. The primary causes of internal aging in silicone rubber were then verified through molecular simulation. A nonlinear evaluation model based on “service years” and “operating altitude” was finally constructed for the rapid and accurate assessment of mechanical properties.

## 2. Test Results and Analysis of Aging Mechanical Properties of Composite Insulation Materials

### 2.1. Sample Preparation

**Figure 1:**
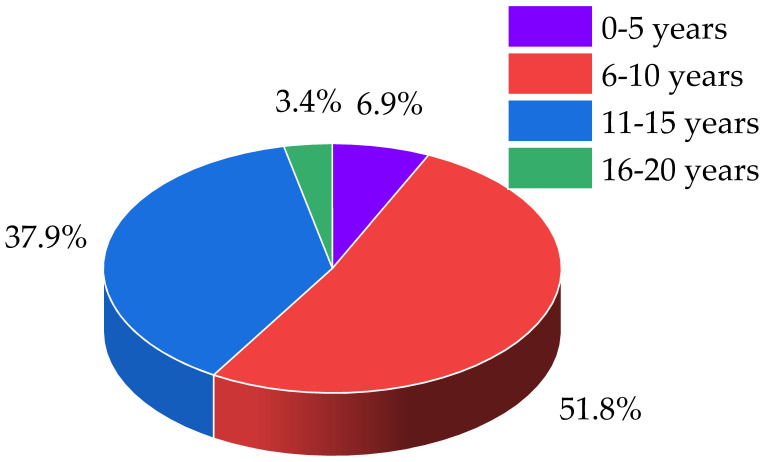
Composite insulation sample statistics.

The composite insulator samples selected for this study were sourced from four typical environments: Tibet (ultra-high altitude with extreme ultraviolet radiation), Yunnan (high altitude with distinct dry and wet seasons), Hunan Xuefeng Mountain (mid-to-high altitude with ice, snow, and lightning), and Anhui/Chongqing (low-to-mid altitude with industrial pollution, heat, and humidity). Surface aging can vary despite similar operating conditions. Aiming for a generalizable model that avoids overfitting, we sampled composite insulators from diverse service conditions and material batches, classifying them by source manufacturer (A–D). Grouping by manufacturer controls for material and process variables, thus enabling a focused analysis of how the natural environment and service time drive performance degradation. Figure 1 presents the operating life distribution and sample proportion breakdown; refer to Table 1 and Table 2 for grouping details. The altitude and service life are represented by *H* and *T*, respectively. It is crucial to distinguish the dual usage of terms such as “D3” and “D4” in this manuscript. In Tables and Figures, these labels are employed to denote sample identifiers (e.g., “D1” represents Sample 1 from Manufacturer D). Conversely, within the discussion on aging mechanisms in the main text, the same notations are used to refer to the low-molecular-weight cyclosiloxane products generated during silicone rubber aging, such as octamethylcyclotetrasiloxane (D4) and hexamethylcyclotrisiloxane (D3).

As shown in Figure 1, 41.3% of the sampled insulators are found to have been in service for over 10 years (11–15 year interval), with a notable concentration in the 6–10 year segment. This distribution profile not only highlights the service-age structure of the in-service insulator population but also underscores the urgent need to calculate the remaining strength of composite insulators.

### 2.2. Aging Characteristics of Samples

**Figure 2:**
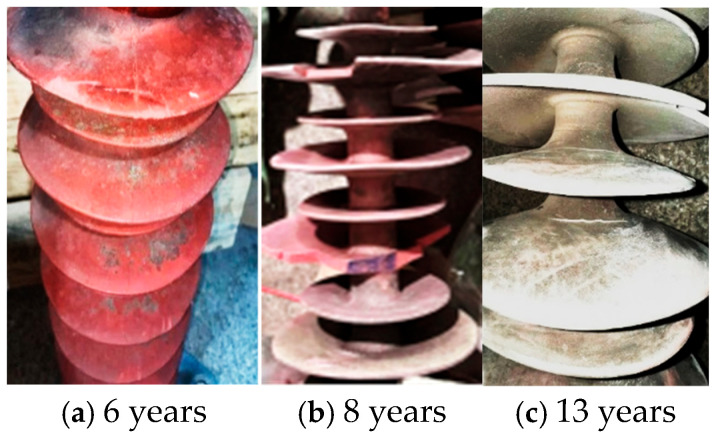
Appearance of composite insulators with different service lives: (**a**) 6 years, (**b**) 8 years, (**c**) 13 years.

According to standard DT810-2012 [16,17,18], pulverization means that the surface of insulator umbrella skirt filler particles is rough or powdery, and cracks are defined as micro-cracks with a depth of 0.01~0.1 mm. Selected samples had different ages and were assessed by appearance inspection, and the aging characteristics were classified according to standards, as shown in Figure 2.

As shown in Figure 2, the aging morphologies of composite insulator sheds after 6, 11, and 13 years of grid service are sequentially characterized as possessing severe surface contamination accompanied by edge wear, significant material embrittlement, hardening, and powdering, and severe cracking at the shed root and edges. These phenomena indicate that the aging process follows a distinct progression: it is initially characterized by surface pollution and wear, gradually develops into performance degradation of the material bulk (embrittlement, hardening, cracking), and ultimately leads to structural failure (cracking, tearing).

### 2.3. Test Methods and Instruments

In order to verify the rationality of the classification of the aging state of samples, the following physical and chemical properties were tested:

(1) Hardness testing was performed using a Shore durometer. Six equally spaced points (6 mm intervals) along the umbrella skirt’s root-to-edge axis were measured with vertical compression. The surface hardness, expressed as the six-point average, quantifies local deformation resistance.
(2) Tensile strength and elongation at break were measured using an EZ-LX universal testing machine. Tensile strength represents the material’s resistance to tensile loads.
(3) Shear strength and deformation coefficient were tested by applying a parallel shear force to the specimen cross-section at a constant loading speed until failure. Shear strength quantifies a material’s resistance to shear failure, defined as the maximum stress per unit area prior to fracture.
(4) Puncture testing was conducted per ASTM D7192 [19], applying vertical loads to specimens with real-time deformation monitoring. The deformation coefficient defines the material’s deformation energy and puncture resistance.

### 2.4. Hardness

**Figure 3:**
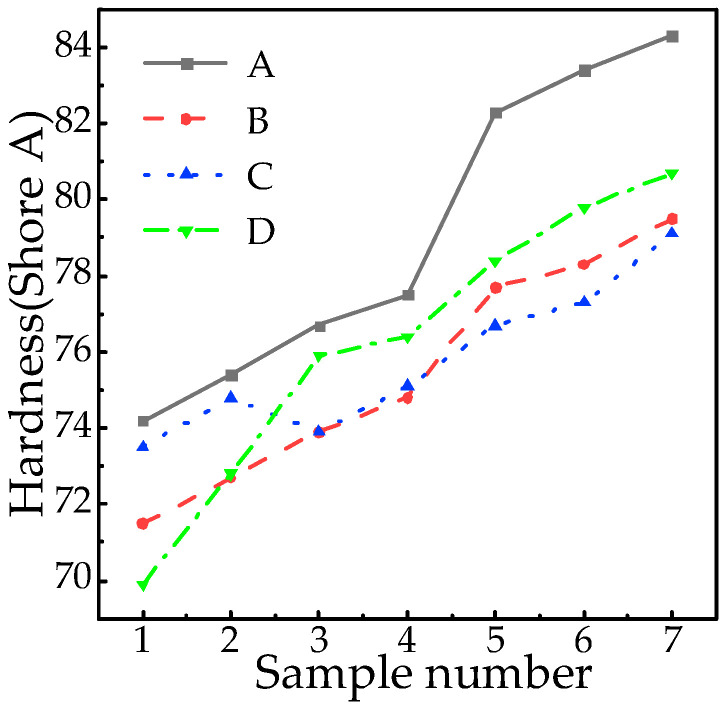
Hardness test results of composite insulations.

In standardized evaluations of silicone rubber aging, hardness is utilized as a key indicator of degradation, reflecting the material’s resistance to localized deformation. A Shore durometer was used to apply vertical loads at six equidistant points (spaced 6 mm apart) along the radial direction of the umbrella skirt to quantify the extent of degradation. The surface hardness, represented by the average of the six measurements, is presented in Figure 3.

Figure 3 demonstrates a consistent surface hardness increase across manufacturers with aging progression (+13.6% A, +11.2% B, +7.62% C, +15.5% D), confirming silicone rubber hardening during degradation. Factory D exhibited the most pronounced change, particularly showing nonlinear acceleration between D1–D3 (D1–D2: +4.1%; D2–D3: +4.3%), potentially linked to cross-linking network heterogeneity in early aging stages. Critically, samples exceeding 80 Shore A (e.g., A7/D7) partially lose elastic deformation capacity, inducing surface embrittlement risks.

### 2.5. Tensile Strength and Elongation at Break

**Figure 4:**
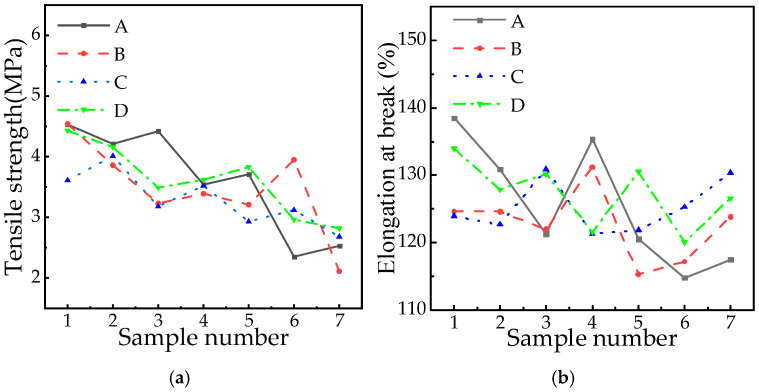
Tensile strength and elongation at break. (**a**) Tensile strength. (**b**) Elongation at break.

Tensile strength serves as the primary mechanical performance indicator for composite insulators, quantifying the silicone rubber material’s resistance to tensile loads. Test results for tensile strength and elongation at break are presented in Figure 4.

Figure 4 reveals non-monotonic variations in tensile strength across manufacturers with service life/altitude. For example, the tensile strength of A1 (167 m above sea level) after six years of operation was 4.53 MPa, while A4 (475 m above sea level) after nine years of operation was reduced to 3.54 MPa, and A6 (4546 m above sea level) was further reduced to 2.35 MPa, showing a general downward trend. However, the tensile strength of B3 (10 years, 2415 m) in plant B reached 3.23 MPa, while that of B6 (15 years, 2415 m) rose to 3.95 MPa. At the same altitude (1450 m), the tensile strength of C1 (7 years) and C2 (9.5 years) in plant C increased to 3.61 MPa, while that of D1 (3.5 years) and D7 (14 years) in plant D decreased to 2.82 MPa. These non-monotonic variations confirm that while localized strength increases occur, tensile strength exhibits predominant degradation with service life extension. In terms of elongation at break, there are data fluctuations in B1 (124.6%) and B4 (131.2%) in plant B, while C1 (123.9%) to C7 (130.4%) in plant C declined as a whole. C3 (130.9%) and C6 (125.3%) rose in the middle, while D1 (134.0%) fluctuated in plant D. It shows that the two mechanical properties may be affected by many other factors.

### 2.6. Shear Strength and Shear Deformation Coefficient

**Figure 5:**
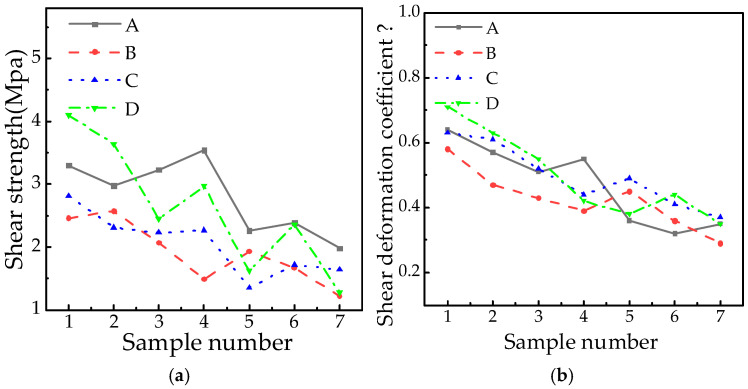
Shear strength and shear deformation coefficient. (**a**) Shear strength. (**b**) Shear deformation coefficient.

Shear strength defines a material’s capacity to resist shear failure under applied stress, representing the maximum shear force per unit area. The shear deformation coefficient (*β*) characterizes material deformation behavior under shear loading. Figure 5 presents the variations in shear strength and coefficient *β* across the tested samples.

Figure 5 shows dispersed strength and deformation coefficient values. Statistical analysis results confirm these properties degrade with extended service duration and higher altitudes. For example, the shear strength of A7 (4546 m, 8 years) at high altitude is 40% lower than that of A1 (167 m, 6 years), and that of B7 (2328 m, 17 years) in B factory is 50.4% lower than that of B1 (1824 m, 6 years), which shows that intermolecular force intensifies with environmental severity. At the same time, there are also nonlinear evolution phenomena in the data: the shear strength of A4 (9 years) in A factory is 9.6% higher than that of A3 (8 years), and that of B2 (8 years) in B factory is 4.5% higher than that of B1 (6 years), which indicates that there are periodic performance fluctuations in the aging process of materials. The shear deformation coefficient *β* generally shows a downward trend with the increase in operating life and altitude, but there are significant fluctuations among different manufacturers and samples, and the decline of the *β* value is intensified in high altitude environments.

### 2.7. Puncture Strength and Puncture Deformation Coefficient

**Figure 6:**
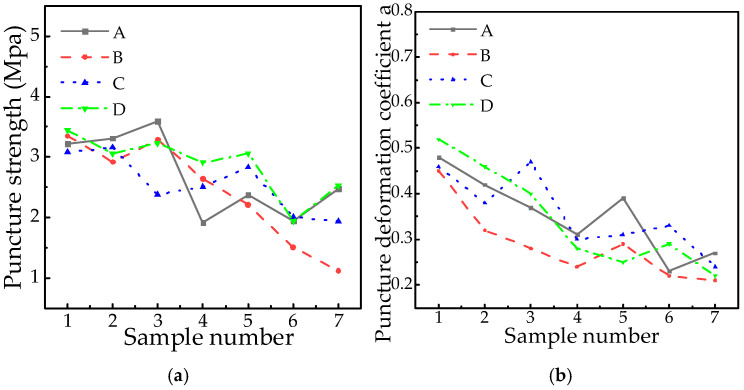
Puncture strength and puncture deformation coefficient. (**a**) Puncture strength. (**b**) Puncture deformation coefficient.

Puncture strength quantifies a material’s resistance to sharp object penetration, reflecting its capacity to withstand impact damage. In silicone rubber, this property depends on hardness-toughness balance, but aging-induced molecular chain fracture, cross-link failure, and load transfer reduction cause atypical hardening and strength attenuation. This degradation cascade eventually leads to the embrittlement of umbrella skirts. Figure 6 presents the puncture strength and deformation coefficient test data.

Figure 6 exhibits significant fluctuations in puncture strength and deformation coefficient. For example, in Factory A, the puncture strength of sample A1 at low altitude (6 years) is 3.22 MPa, while that of sample A6 at high altitude (9 years, 4546 m) is 1.94 MPa but that of sample A3 (8 years, 319 m) is 3.59 MPa, which indicates that the local stress state or the change in material microstructure may cause a fluctuation in puncture performance. The puncture strength of B1 (6 years) and B7 (17 years) in plant B decreased to 1.12 MPa, with an average annual decrease of 3.35 MPa, but B3 (10 years) increased by 12.7% compared with B2 (8 years). These non-monotonic patterns confirm that puncture performance cannot be uniformly predicted by service years or altitude, likely due to localized stress states and micro-structural evolution.

The puncture deformation coefficient α exhibits an overall decreasing trend with extended service years and higher altitudes, yet demonstrates significant fluctuations. For instance, the α value of high-altitude samples (A6, 4546 m above sea level, α = 0.23) is generally lower than that of similar samples at low altitude, but the α value of some long-term samples (such as B5, running for 13 years) is higher than that of some short term samples (B4, running for 11 years). These variations may originate from localized cross-link recombination or sampling position heterogeneity, confirming that the deformation coefficient *α* alone cannot sufficiently characterize material degradation.

### 2.8. Analysis and Discussion

Based on the experimental results from Section 2.4, Section 2.5, Section 2.6 and Section 2.7, the key mechanical parameters of composite insulators including tensile, puncture, and shear strength exhibit no clear monotonic correlation with service duration or altitude. This nonlinear aging behavior originates from the complex synergistic interplay between multiple stressors in the actual operating environment, such as salt-alkali corrosion, high-altitude UV radiation, and pollutant deposition, and intrinsic material properties, including filler characteristics and manufacturing processes. Although the overall degradation trend aligns with findings reported in the existing literature [7,8], the degree of non-linearity revealed in this work is significantly more pronounced than results typically observed in controlled, single-factor laboratory accelerated aging tests [6]. This discrepancy directly confirms that coupled multi-factor conditions encountered in real-world service induce far more complex aging mechanisms. Consequently, conventional assessment methods that rely solely on a single mechanical indicator are inadequate for accurately characterizing such behavior. This further underscores the importance of investigating internal degradation mechanisms through molecular simulation and the imperative need to establish a comprehensive characteristic parameter capable of integrally reflecting the influence of multiple factors.

## 3. Simulation of Mechanical Property Degradation in HTV Silicone Rubber Composites

### 3.1. Construction of Sample Models

This study focuses on a composite insulator with key components made of high-temperature vulcanized (HTV) silicone rubber, which is based on methyl vinyl silicone rubber (MVQ). This study employed high-temperature vulcanized (HTV) silicone rubber based on methyl vinyl silicone rubber (MVQ) to construct a model for composite insulators. Due to the difficulty in directly modeling silicone rubber with high molecular weight (400,000–800,000), and although the degree of polymerization has a limited impact on simulation results [20,21], we selected the model configuration with the highest vinyl content (corresponding to a polymerization degree of 64) to preserve the fundamental material properties and ensure sufficient crosslinking strength [22,23]. Based on the crystalline SiO_2_ structure, a nanocluster with a radius of 7.5 Å was constructed using the Build Nanocluster module in Materials Studio 2023. A composite model with an initial density of 0.6 g/cm^3^ was built using polydimethylsiloxane (PDMS) and the crosslinking agent DBPMH at a molar ratio of 1:1 (each containing eight molecular chains), and energy minimization was performed under the COMPASS III force field [24]. Periodic boundary conditions were applied in this study to simulate the bulk behavior of an unbounded macroscopic material, ensuring the correctness of the thermodynamic ensemble and computational feasibility. After setting the relevant parameters, the simulation was run to complete the encapsulation of methyl-vinyl silicone rubber (MVQ) molecules. The initial model temperature was set to 300 K. The crosslinking process (including annealing for both heating and cooling) was triggered at 383 K and continued to 438 K, with a reaction radius of 4–18 Å and a target crosslinking degree of 100%. Through annealing and dynamic simulation, the positions of molecular chains were gradually adjusted until active atoms fell within the reaction radius, either achieving the target degree or exceeding the maximum radius. Crosslinking was achieved by the breaking of O–O peroxide bonds in DBPMH and their subsequent reaction with vinyl groups in the PDMS main chains. Due to the size limitations of the amorphous model and the search radius, the maximum crosslinking degree attained was 86.7%. The system was then cooled stepwise at 20 K intervals with 200 ps relaxation per step. After reaching 363 K, it was equilibrated in the NPT ensemble for 200 ps to ensure stable energy, density, and structure.

**Figure 7:**
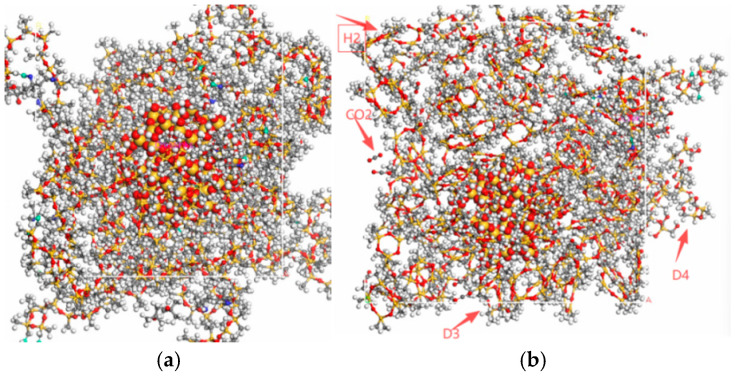
MVQ system modes. (**a**) MVQ-1. (**b**) MVQ-3.

According to the XPS elemental analysis of composite insulator silicone rubber at different aging stages reported in the literature [25], SiO_2_/MVQ composite models corresponding to various aging states were developed by incorporating aging-generated by-products. Although XPS primarily detects surface layers up to 10–15 nm in depth, and its signals may be affected by surface-adsorbed species, the samples in the cited literature were pretreated with ethanol cleaning, which effectively minimized contamination. Therefore, with appropriate sample handling, XPS data can offer meaningful surface information indicative of the material’s overall chemical evolution. To preserve the global elemental mass balance and regulate the Si, O, and C contents in the silicone rubber matrix, the influences of aging by-products (D_4_, D_3_, CO_2_, CH_4_, and H_2_) were considered. Accordingly, they were introduced with the following quantities: 50 D4, 60 D3, 25 CH_4_, 20 H_2_, and 36 CO_2_ in the MVQ-3 system, and 60 D4, 56 D3, 20 CH_4_, 25 H_2_, and 40 CO_2_ in the MVQ-5 system. Based on elemental analyses at different aging stages and the identified by-products, molecular models corresponding to samples C1, C3, and C5 were established in Materials Studio using Theodorou’s polymer modeling approach [26] and designated as MVQ-1, MVQ-3, and MVQ-5 (compositions listed in Table 3). By integrating MVQ molecular chains with the corresponding aging by-products, three systems with silicon contents of 36.36%, 35.35%, and 35.16% were constructed. Among them, D4, D3, and other by-products were introduced into the MVQ-3 and MVQ-5 models. Schematic diagrams of the MVQ-1 and MVQ-3 models are shown in Figure 7.

### 3.2. Molecular Dynamics Equilibrium Simulation

**Figure 8:**
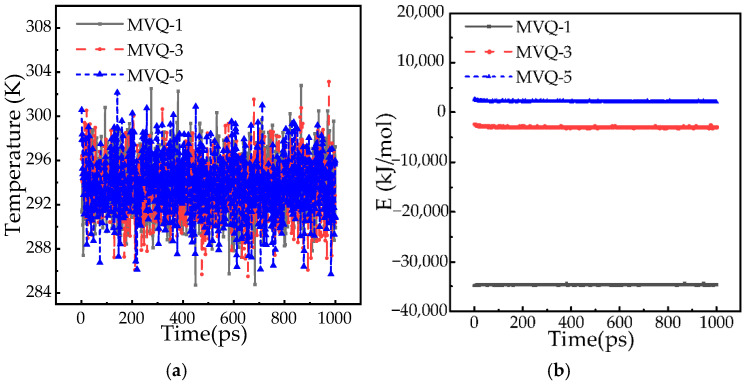
Energy and temperature of the MVQ-3 system. (**a**) Temperature. (**b**) Total energy.

Prior to molecular dynamics simulation, the MVQ model is optimized and annealed using the Forcite module. The simulation is conducted at 363 K and 101 kP. The velocity Verlet algorithm is employed to solve the equations of motion, while van der Waals and electrostatic interactions are handled using the atom-based and Ewald methods, respectively [27,28]. Temperature and pressure are controlled by the Nose and Berendsen thermostat methods, with the COMPASSIII force field assigned. The molecular dynamics simulation is subsequently performed under the canonical ensemble (NVT) for a duration of 1000 ps [29]. All calculations are completed within the MS 2023 software package. The temperature and energy convergence curves of the optimized model are presented in Figure 8.

The lowest energy value is found in the MVQ-1 system. Under the same force field, stability is governed by the energy magnitude rather than its sign, with lower energy indicating higher stability. Consequently, the MVQ-1 system is regarded as the most stable. As the aging of the MVQ system becomes more severe, the total system energy is increased, and its stability is correspondingly reduced. After the three aged MVQ systems reached steady-state equilibrium, the fluctuation of the total system energy was consistently maintained within 0.3%. According to thermodynamic criteria, a molecular structure is considered stable when the energy variation during molecular simulation is kept between 1% and 5%. Therefore, the optimized molecular structure is considered to be in a stable state. Moreover, under constant temperature conditions maintained at 298 K, it is observed that with an increasing number of simulation steps, the energy of the highly aged MVQ model gradually approaches equilibrium [30], which further confirms the reliability of the model.

### 3.3. Mechanical Performance Analysis

**Figure 9:**
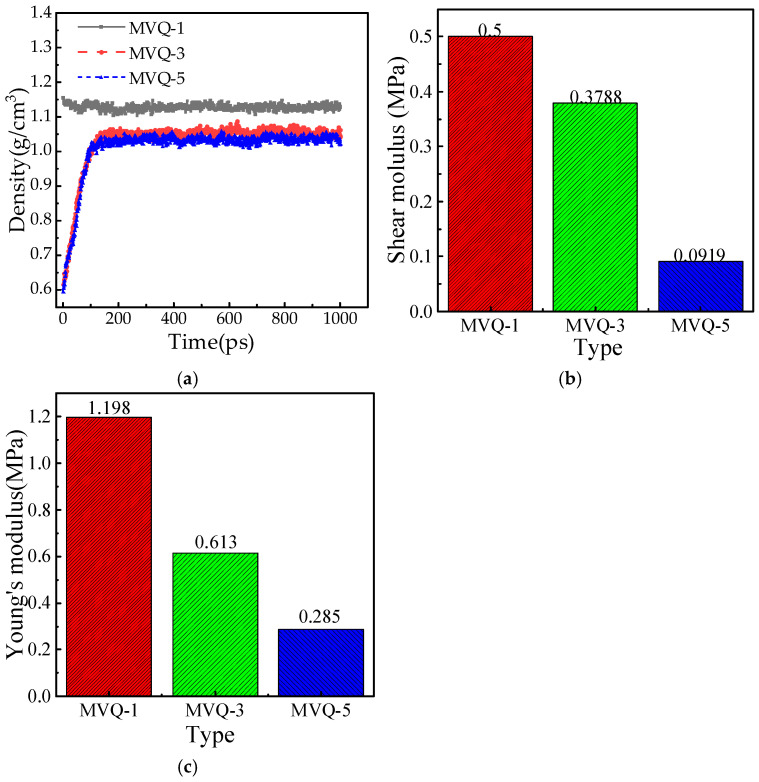
Different MVQ systems. (**a**) Density. (**b**) Shear modulus. (**c**) Young’s modulus.

Shear modulus (*G*) is an index to measure the ability of materials to resist shear deformation, and elastic modulus (*E*) usually refers to Young’s modulus, which describes the ability of materials to resist elastic deformation (that is, recoverable deformation). First, density analysis was performed on the dynamically equilibrated models using the Analysis tool in Materials Studio, and the resulting density data were exported to generate Figure 9a. Subsequently, modulus parameters were calculated using the Mechanical Properties module of the Forcite software. The last six frames of the trajectory file were selected, the maximum strain amplitude was set to 0.003, and the number of steps per strain was set to four, from which the corresponding Lamda (λ) and *μ* values were obtained. The shear modulus G and Young’s modulus *E* satisfy the following relationships: *G* = *μ*, and *E* = *μ*(2*μ* + 3*μ*)/(*λ* + *μ*). The shear modulus and Young’s modulus of the SiO_2_/MVQ composites at three different aging stages are presented in Figure 9b,c.

As shown in Figure 9, with the intensification of the aging process, the overall density of the nano-SiO_2_/MVQ composite system continuously decreases, but the rate of decline gradually slows. This delay in density reduction may be attributed to the cross-linking reactions within the silicone rubber molecules [31,32]. At the severe aging stage, both the shear modulus and elastic modulus of the nano-SiO_2_/MVQ system decrease significantly, exhibiting a distinct nonlinear declining trend. This phenomenon is likely closely related to the rapid decrease in silicon content and the continuous increase in oxygen content within the system [33]. These findings provide a theoretical basis from a simulation perspective for understanding the aging mechanism of silicone rubber materials.

### 3.4. Significance and Limitations of Molecular Simulation in the Present Research

This study established a system of microscopic aging characteristic parameters by integrating simulation and experimental data, with the core parameters including the number of broken bonds in molecular chains, key small-molecule products, and the relative contents of silicon/carbon elements [34,35]. Based on this, molecular models reflecting different degrees of aging were constructed and simulation calculations were performed. The calculation results clearly demonstrated a systematic decline in the macroscopic mechanical properties of the models as aging progressed, with all performance parameters of the deeply aged model being the lowest. This directly confirms that the accumulation of micro-structural damage is the fundamental cause of macroscopic mechanical performance degradation. The core significance of this work lies in the establishment of a quantifiable link that directly connects experimentally observable microscopic chemical features (such as bond breakage and product) with the macroscopic mechanical properties that determine the service safety of insulators. This provides a coherent picture from the atomic to the macroscopic scale for understanding the aging failure of composite materials and demonstrates the potential of using microscopic indicators to predict macroscopic performance degradation. However, certain limitations of this method are acknowledged: firstly, the mapping relationship from a limited set of microscopic features to complex macroscopic behavior may be oversimplified; secondly, necessary simplifications were made regarding the mechanisms of certain complex chemical reactions (such as competitive reactions involving multiple free radicals) in the simulations; finally, extrapolating the trends from microscopic models to the lifetime prediction of real components still requires validation across multiple scales.

## 4. Prediction Method of Aging Mechanical Strength of Composite Insulation Materials

### 4.1. Rapid Prediction of Composite Insulator Aging Based on Service Years and Altitude

To quantify the relationship between the aging state and mechanical strength of composite insulators, a rapid prediction method was developed based on experimentally measured data. First, the service years and altitude distribution of the test samples were statistically analyzed, followed by tensile, puncture, and shear strength tests. The results revealed a significant synergistic effect between the cumulative influence of service years and altitude. To characterize this synergy, normalization was performed using 20 years of service and 5000 m altitude as reference values, corresponding to the actual operating conditions of the samples. The normalized data were then fitted to a multi-factor interaction model, and the model parameters a, b, and c were determined through regression analysis. Consequently, a characteristic aging parameter relationship *Q*(*T*, *H*) was established, comprehensively reflecting the synergistic effects of service years and altitude. The predictive accuracy of this model will be further validated through subsequent experimental data.

### 4.2. Analysis of Aging Characteristic Quantity of Mechanical Properties

Molecular dynamics (MD) simulations were conducted based on existing aging research to construct microstructural models representing different aging stages and to systematically validate the evolution of mechanical properties after aging. Through this approach, the atomic-scale mechanisms responsible for mechanical degradation during aging were revealed, including main chain scission, a decrease in system density, and changes in the elemental chemical environment. Building on this foundation, the Ogden hyper-elastic constitutive model is employed in this section to translate the aforementioned microscopic mechanisms into a quantitative description and prediction of the evolution of macroscopic stress–strain responses. Therefore, an overall mechanical evaluation metric for composite insulating components is proposed in this section. This metric integrates characteristic energy parameters from the three crack evolution stages in silicone rubber sheds—initiation, stable propagation, and unstable fracture—with the theories of fracture energy release rate, a viscoelastic damage model, and aging kinetics equations. First, according to hyper-elastic constitutive theory (the Ogden model) [36], the strain energy density of the material is determined by both tensile strength and elongation at break. The large-deformation behavior of silicone rubber materials is described by a multi-modal strain energy function, with its constitutive equation expressed as follows:

(1) $$W=\sum_{i=1}^{N}\frac{\mu_{i}}{\alpha_{i}}\left(\lambda_{1}^{\alpha_{i}}+\lambda_{2}^{\alpha_{i}}+\lambda_{3}^{\alpha_{i}}-3\right),$$

(2) $$W_{failure}=\int_{0}^{\lambda_{b}}\sigma \left(\lambda \right)d\lambda \approx \sigma_{t}\cdot \left(\lambda_{b}-1\right),$$

where $\lambda_{b}-1$ is the elongation ratio at break. J-integral is a parameter representing the energy release rate of the crack tip in fracture mechanics, which reflects the energy required to be released per unit area of crack propagation, as shown in the following formula:

(3) $$J=\int_{\Gamma }\left(Wdy-T\cdot \frac{\partial u}{\partial x}ds\right).$$

*W* is strain energy density, *T* is surface force vector and *u* is displacement field. The shear lag model describes the relationship between shear stress and crack propagation through energy release rate, and describes that energy is released during crack propagation [37], as shown in the following formula:

(4) $$\left\{\begin{array}{l} G=\frac{{\tau_{q}}^{2}\cdot \Delta l}{2G_{s}}\\ \beta =\frac{\Delta l}{L} \end{array}\right.\to \sqrt{G}\propto \tau_{q}\cdot \sqrt{\beta },$$

where *G* is the energy release rate of crack propagation, $\tau_{q}$ is the shear strength, Δl is the crack propagation length, and $G_{s}$ is the shear modulus. Log function is introduced as a hardness-energy absorption inhibitor to form a load energy absorption term considering embrittlement effect, as follows:

(5) $$\left(\frac{\sigma_{t}\cdot \varepsilon_{b}}{\log\left(1+H_{a}\right)}\right),\left(\frac{\delta_{p}\cdot \sqrt{\alpha }}{\log\left(1+H_{a}\right)}\right),\left(\frac{\tau_{q}\cdot \sqrt{\beta }}{\log\left(1+H_{a}\right)}\right).$$

Based on Thai-Hill multi-axial failure criterion and simplifying its quadratic term, the linear superposition analysis of the synergistic effect of stretching, shearing and puncture can be obtained:

(6) $$D=\left(\frac{\sigma_{t}\cdot \varepsilon_{b}}{\log\left(1+H_{a}\right)}+\frac{\delta_{p}\cdot \sqrt{\alpha }}{\log\left(1+H_{a}\right)}+\frac{\tau_{q}\cdot \sqrt{\beta }}{\log\left(1+H_{a}\right)}\right)\cdot \log(1+\varepsilon_{b}).$$

The aging characteristic quantity *Q* is defined to quantify degradation severity through the energy absorption coefficient *D*. Consistent with reference [38], *D* exhibits an inverse relationship with aging extent (higher *D* indicates less degradation). The proposed relationship between *Q* and *D* is:

(7) Q=exp(−D).

The aging characteristic quantity *Q* can be expressed as the following formula, where α is defined as the deformation coefficient after crack propagation:

(8) $$Q=\exp\left(-\frac{\sigma_{t}\cdot \varepsilon_{b}+\delta_{p}\cdot \sqrt{\alpha }+\tau_{q}\cdot \sqrt{\beta }}{\log\left(1+H_{a}\right)}\cdot \log(1+\varepsilon_{b})\right).$$

### 4.3. Microscopic Interpretation of the Aging Characteristic Q

The macroscopic mechanical aging characteristic *Q*, defined in Equation (8), is structurally interpreted as follows: its numerator integrates the material’s energy absorption capacity across tensile, puncture, and shear loads during crack initiation and propagation, its denominator characterizes the embrittlement effect from increased hardness, and a logarithmic term corrects for the material’s elastic ductility. Given its critical role, a systematic analysis of the factors contributing to *Q* is conducted and correlated with chemical changes in rubber composites under actual aging, extending beyond simplified molecular dynamics models. It is demonstrated that the *Q* value exhibits a significant increasing trend with extended service duration or elevated altitude. As a composite indicator coupling tensile, shear, puncture, and hardness properties, *Q* integrates energy absorption, embrittlement, and ductility correction to quantitatively characterize complex multi-factor interactions. Its regular variation indicates that *Q* overcomes the limitations of single-parameter indicators [39], systematically reflecting the mechanical aging process in complex service environments. From an energy perspective, *Q* quantifies the mechanical aging degree of composite insulating components, effectively capturing the cumulative effect of service time and the synergistic influence of altitude-based environmental conditions, thereby providing a comprehensive and reliable quantitative basis for aging state assessment [8,40].

### 4.4. Calculation of Aging Characteristic Quantity of Mechanical Properties

**Figure 10:**
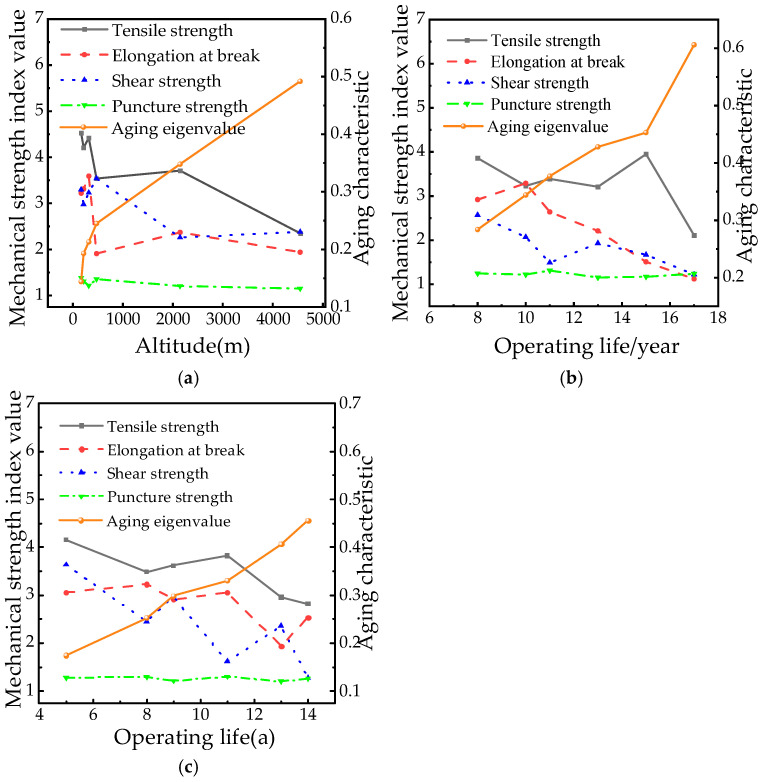
Characteristic quantity *Q* of each factory. (**a**) Factory A. (**b**) Factory B. (**c**) Factory D.

To quantify the correlation between the aging state and mechanical strength of composite insulators, the service years, altitude distribution, and mechanical test results (tensile, puncture, and shear strength) obtained from Section 2.1, Section 2.2, Section 2.3, Section 2.4, Section 2.5, Section 2.6, Section 2.7 and Section 2.8 were analyzed. A significant synergistic effect between the cumulative influence of service years and altitude was identified. Quantitative aging indicators were subsequently obtained by substituting the normalized data into Equation (8). Taking Manufacturer *D* as an example, the calculated performance indicators and corresponding *Q* values are presented in Table 4. Furthermore, the evolution trends of mechanical properties and *Q* values with service years for manufacturers A, B, and D are illustrated in Figure 10.

Table 4 data confirm that material aging reduces energy absorption capacity while increasing *Q* values, directly reflecting mechanical degradation from an energy perspective. Critically, *Q* demonstrates a consistent upward trend with extended service years and higher operational altitudes, establishing a quantifiable aging progression pattern. Service life exhibits dual-phase degradation: during years 0–8, physical aging predominates, driving tensile property reduction at 4.1% annually while elevating *Q* values. After more than 10 years, the cross-linking density increased, the hardness embrittlement term increased by 1.8% annually, and the *Q* value increased by 0.045 per year. For example, from D3 (8 years) to D7 (14 years) in the D plant, the *Q* value jumped from 0.253 to 0.456, and the hardness increased from 75.9 HA to 80.7 HA simultaneously. High-altitude exposure (4546 m) elevates hardness embrittlement by 5.2% (0.341) and reduces puncture resistance by 33.4% (0.221) versus 1450 m baselines. These metrics confirm accelerated crack initiation, intensified cross-linking, and enhanced embrittlement under extreme conditions.

As observed in Figure 10, the aging characteristic quantity *Q* is found to exhibit a significant upward trend with increasing service years and altitude. This behavior is attributed to *Q*’s integration of tensile, shear, and puncture properties, coupled with the fusion of material energy absorption capacity and brittleness enhancement (hardness term), including extension corrections. These integrations enable the quantification of multi-factorial degradation effects. Crucially, *Q*’s systematic progression is demonstrated to overcome single-parameter limitations by comprehensively reflecting mechanical aging in complex environments. The synergistic effects of temporal accumulation and altitude exposure are thus reliably quantified, establishing a robust quantitative basis for aging state evaluation.

### 4.5. Influence of Superposition of Multi-Factor Variables on Characteristic Quantity

In order to further explore the specific functional relationship of *Q* ∝ *f*(*T*, *H*), the transformation of *Q* expression shows that:

(9) $$Q=E\cdot \exp\left(A+B+C\right).$$

A single variable is positively correlated with the *Q* value, and the exponential function relation of the aging characteristic quantity *Q* is put forward. Let *Q*(*T*, *H*) satisfy the following formula:

(10) $$Q=d\cdot \exp\left(f\left(T,H\right)\right).$$

Further, multi-factor interaction modeling is used; *f*(*T*, *H*) should satisfy the following formula:

(11) $$f\left(T,H\right)=a\cdot T+b\cdot H+c\cdot T\cdot H.$$

Therefore, *Q*(*T*, *H*) should satisfy the following formula:

(12) $$Q=d\cdot \exp\left(a\cdot T+b\cdot H+c\cdot T\cdot H\right).$$

**Figure 11:**
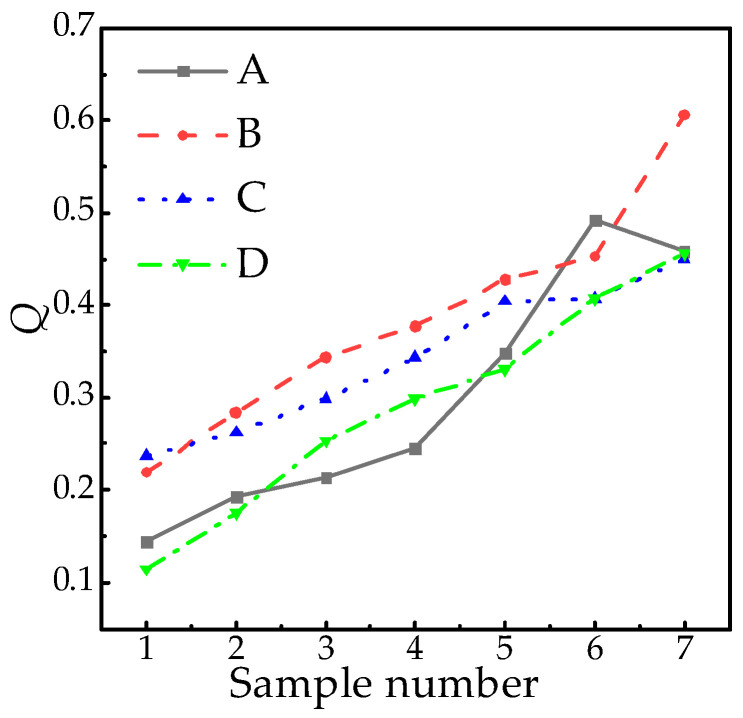
Conventional residual diagram.

Based on the actual service years and altitude of the test samples, normalization was performed using 20 years and 5000 m as reference values. The normalized data were then substituted into the formula derived from multi-factor interaction modeling, and the parameters a, b, and c were determined through regression analysis. Consequently, the relationship *Q*(*T*, *H*) was established. Representative cases from Factories A and B are presented in Table 5. The normalized parameter diagram is shown in Figure 11.

Substituting the data in Table 6 into Equation (12) for fitting, the calculated coefficients a = 1.62, b = 0.9, c = 0, d = 0.1, and *Q*(*T*, *H*) are as follows:

(13) Q=0.1exp(1.62T+0.9H).

**Figure 12:**
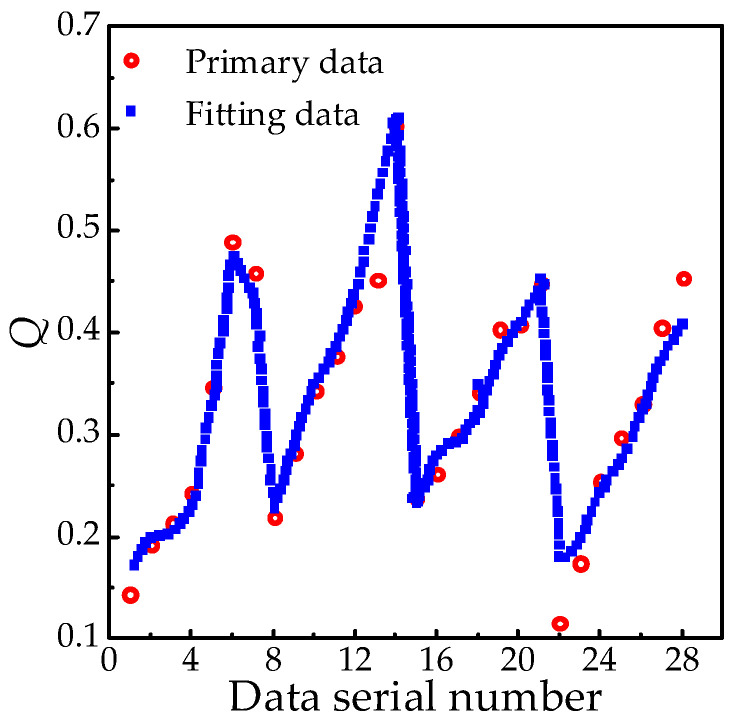
Characteristic quantity *Q* of mechanical properties.

**Figure 13:**
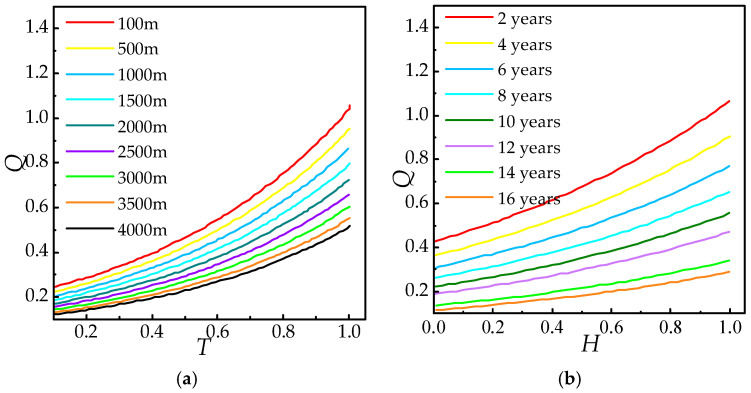
Function *Q*(*T*, *H*) surface and graph. (**a**) Influence of operating life *T*. (**b**) Influence of operating altitude *H*.

The fitting function’s coefficient of determination (R2), root mean square error (RMSE), and mean absolute error (MAE) are presented in Table 6. Conventional residual plots and fitting comparison diagrams are further illustrated in Figure 12. Figure 13 displays the characteristic surface and functional relationship of *Q*(*T*, *H*). 

Judging from the error index, the determination coefficient R2 (0.9527) is close to 1, which shows that the fitting function has strong explanatory power regarding the variation in *Q* value, and the independent variables (age factor *T* and altitude factor *H*) can effectively describe the variation law of *Q*. With RMSE and MAE, both error metrics demonstrate minimal deviation between predicted and actual values, confirming high predictive accuracy.

The zero cross-term coefficient confirms that *f* (*T*, *H*) exhibits strict additivity: service duration (*T*) and altitude (*H*) independently influence composite insulator aging without synergistic enhancement or inhibition, resulting in purely super-positional effects. From the univariate mechanism, the coefficient of operating years *T*(1.62) is significantly greater than that of the altitude factor *H*(0.9), which indicates that operating years have a stronger driving effect on the *Q* value under the framework of the exponential growth model. This difference reflects the dominance of the time accumulation effect (such as the increase in cross-linking density and the decrease in crack growth threshold) in the aging process of composites, while a high-altitude environment (ultraviolet radiation and low-pressure oxidation) further promotes the increase in *Q* value by accelerating chemical degradation.

### 4.6. Rapid Prediction Method and Verification of Mechanical Strength

From the point of view of energy, the characteristic quantity *Q* of mechanical property aging of composite insulation parts is put forward. The exponential function relationship between the characteristic quantity *Q* and years and altitude is as follows:

(14) $$f\left(p\right)=b\cdot \exp\left(\pm K\cdot t^{\gamma }\right),$$

where *f*(*p*) is the ratio of aging performance to initial aging performance; *K* is the velocity coefficient; *B* and γ are constants; *T* is the aging time. The strength threshold of composite material is defined as *x*, and the relationship between the material strength threshold *x* and the characteristic quantity *Q* combined with Formula (14) may satisfy the following relationship:

(15) $$Q=e\cdot \exp\left(-g\cdot x^{\omega }\right).$$

**Figure 14:**
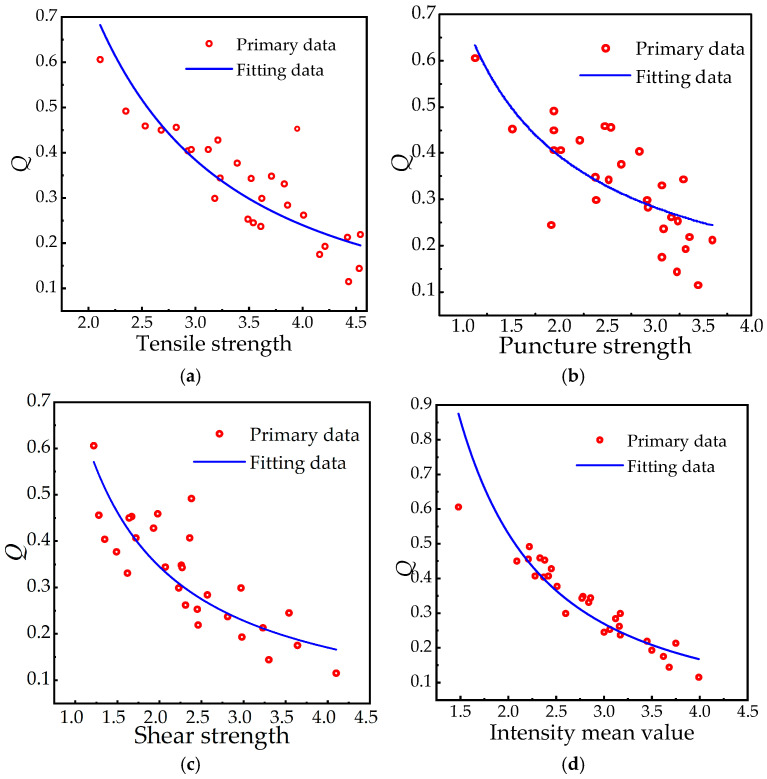
*Q* value, comparison chart of strength test values, and fitting curve. (**a**) Tensile strength fitting. (**b**) Puncture strength fitting. (**c**) Shear strength fitting. (**d**) Intensity mean fitting.

In the formula, *e*, *g* and *ω* are constant coefficients, and the strength threshold of materials is divided into tensile strength, puncture strength and shear strength due to different load types. At the same time, the functional relationship between aging characteristic quantity and material strength is established, as follows:

(16) Q=1.5exp(−0.55x)

where *x* represents the average strength threshold value of materials (tensile strength, puncture strength, and shear strength) in MPa. Equations (15) and (16) treat the characteristic quantity *Q* as an intermediate variable. These equations enable the prediction of a sample’s material strength x for a defined operational lifetime *T* and altitude *H* in its service environment. Therefore, using the mechanical performance test data from the Section 2, the strength threshold and average values were fitted. The results are shown in Table 7 and Figure 14, which compares *Q* values, strength test data, and fitting curves, respectively.

Figure 14a–d reveals high nonlinear correlations between the aging characteristics and multidimensional mechanical properties (tensile, puncture, and shear strength) of silicone rubber. The 0.9222 correlation coefficient between average strength and characteristic quantity *Q* confirms strong agreement with theoretical model predictions of time-dependent property decay. This dose-effect relationship consistency establishes mechanical properties as scientifically valid aging-state parameters while revealing synergistic response mechanisms among strength parameters during material deterioration. Existing methods for evaluating composite insulator aging—such as hyperspectral imaging, infrared thermal imaging, and traditional parameter-based approaches—are hindered by several limitations, including their inability to capture bulk material properties, susceptibility to environmental interference, weak correlation with mechanical performance, and reliance on empirical fitting. In contrast, the rapid prediction method proposed in this study, grounded in mechanical strength, enables the simultaneous quantification of the synergistic effects of service years and altitude for the first time. The high correlation coefficient of 0.9222 further substantiates the reliability and predictive capability of this approach.

## 5. Conclusions

In this study, the mechanical properties of composite insulation samples from different regions and with varying years of service were analyzed. The following conclusions were drawn:

- Based on existing research, molecular dynamics simulations were employed to construct microstructural models at different aging stages, and it was verified that main chain scission, reduced system density, and changes in the elemental chemical environment during aging are closely related to the degradation of material mechanical properties.
- Macroscopic tests showed that individual strength indicators—such as tensile, puncture, and shear strength—lack monotonic correlations with service years or altitude, attributed to nonlinear aging behavior induced by the synergistic effects of multiple environmental factors. To comprehensively capture these coupled influences, an aging characteristic parameter *Q* was proposed, effectively integrating microstructural evolution with macroscopic mechanical responses.
- A macroscopic strength degradation prediction model centered in *Q* was established as *Q* = 1.5exp(−0.55x). Through the relationship *Q* = 0.1exp(1.62*T* + 0.9*H*), the synergistic effects of service years (*T*) and altitude (*H*) were quantified simultaneously for the first time, with time accumulation confirmed as the dominant aging factor. This model provides both a theoretical foundation and a practical tool for the non-stop rapid strength assessment of insulators in the field.

## Figures and Tables

**Table 1:** Sample factories from A and B factories.

Sample	*T*/a	*H*/m	Sample	*T*/a	*H*/m
A1	6	167	B1	6	1824
A2	8	215	B2	8	2328
A3	8	319	B3	10	2415
A4	9	475	B4	11	2415
A5	10	2145	B5	13	2328
A6	9	4546	B6	15	2415
A7	8	4546	B7	17	2328

**Table 2:** Sample factories from C and D factories.

Sample	*T*/a	*H*/m	Sample	*T*/a	*H*/m
C1	7	1450	D1	3.5	1450
C2	9.5	1450	D2	5	1450
C3	10	1450	D3	8	1450
C4	11	1450	D4	9	1450
C5	13	1450	D5	11	1450
C6	14	1450	D6	13	1450
C7	15	1450	D7	14	1450

**Table 3:** Content of some elements in the silicone rubber matrix.

Pilot Sample	Number	Main Element Content		
		Si	C	O
C1	MVQ-1	36.36%	29.58%	26.71%
C3	MVQ-3	35.35%	28.36%	28.97%
C5	MVQ-5	35.16%	27.84	29.30%

**Table 4:** *Q* and performance indexes of mechanical properties of D factory samples.

Sample	Tensile Property	Puncture Property	Shear Property	*E_b_*	*Q*
D1	0.593	0.496	0.691	0.369	0.115
D2	0.532	0.415	0.578	0.358	0.175
D3	0.454	0.409	0.363	0.362	0.253
D4	0.440	0.308	0.385	0.346	0.299
D5	0.500	0.306	0.200	0.363	0.331
D6	0.355	0.209	0.313	0.343	0.407
D7	0.357	0.237	0.151	0.355	0.456

**Table 5:** Normalized life and altitude values of the samples.

Sample	*T*/a	*H*/m	Sample	*T*/a	*H*/m
A1	0.300	0.033	B1	0.300	0.365
A2	0.400	0.043	B2	0.400	0.466
A3	0.400	0.064	B3	0.500	0.483
A4	0.450	0.095	B4	0.550	0.483
A5	0.500	0.429	B5	0.650	0.466
A6	0.450	0.909	B6	0.750	0.483
A7	0.400	0.909	B7	0.850	0.466

**Table 6:** Calculation error value of fitting function.

Type	Value
R2	0.9527
RMSE	0.0251
MAE	0.0184

**Table 7:** Fitting results of each strength.

Fitting Type	Fitting Formula	R2	RMSE	MAE
Tensile strength	*Q* = 1.5exp(−0.44x)	0.7559	0.0571	0.0421
Shear strength	*Q* = 0.84exp(−0.41x)	0.6912	0.0642	0.0515
Intensity mean value	*Q* = 1.5exp(−0.55x)	0.9222	0.0323	0.0278

## Data Availability

The original contributions presented in the study are included in the article, further inquiries can be directed to the corresponding author.
